# Exploring the potential of a newly developed pectin-chitosan polyelectrolyte composite on the surface of commercially pure titanium for dental implants

**DOI:** 10.1038/s41598-023-48863-2

**Published:** 2023-12-14

**Authors:** Mohammed Husssein M. Alsharbaty, Ghassan A. Naji, Sameh S. Ali

**Affiliations:** 1 Department of Prosthodontics, College of Dentistry, University of Baghdad, Baghdad, Iraq; 2 Branch of Prosthodontics, College of Dentistry, University of Al-Ameed, Karbala, Iraq; 3 College of Dentistry, The Iraqia University, Baghdad, Iraq; 4 School of the Environment and Safety Engineering, Biofuels Institute, Jiangsu University, Zhenjiang, 212013 China; 5 Botany Department, Faculty of Science, Tanta University, Tanta, 31527 Egypt

**Keywords:** Biotechnology, Microbiology, Medical research

## Abstract

Pectin and chitosan are natural polysaccharides obtained from fruit peels and exoskeletons of crustaceans and insects. They are safe for usage in food products and are renewable and biocompatible. They have further applications as wound dressings, body fat reduction, tissue engineering, and auxiliary agents in drug delivery systems. The healing process is usually long and painful. Adding a new material such as a pectin-chitosan composite to the implant surface or body would create unique biological responses to accelerate healing and delivery of target-specific medication at the implant site. The present study utilized the electrospraying process to create pectin-chitosan polyelectrolyte composite (PCPC) coatings with various ratios of 1:1, 2:1, 1:2, 1:3, and 3:1 on commercially pure titanium substrates. By means of FESEM, AFM, wettability, cross-cut adhesion, and microhardness were assessed the PCPC coatings’ physical and mechanical properties. Subsequently, the antibacterial properties of the coating composite were assessed. AFM analysis revealed higher surface roughness for group 5 and homogenous coating for group 1. Group 3 showed the lowest water contact angle of 66.7° and all PCPC coatings had significantly higher Vickers hardness values compared to the control uncoated CpTi samples. Groups 3 and 4 showed the best adhesion of the PCPC to the titanium substrates. Groups 3, 4, and 5 showed antibacterial properties with a high zone of inhibitions compared to the control. The PCPC coating's characteristics can be significantly impacted by using certain pectin-chitosan ratios. Groups 3 (1:2) and 4 (1:3) showed remarkable morphological and mechanical properties with better surface roughness, greater surface strength, improved hydrophilicity, improved adhesion to the substrate surface, and additionally demonstrated significant antibacterial properties. According to the accomplished in vitro study outcomes, these particular PCPC ratios can be considered as an efficient coating for titanium dental implants.

## Introduction

Pectin is an anionic natural polysaccharide that is extracted from plant tissues and widely utilized in the biotechnology and medicinal fields due to its capability to form a gel, thicken, and stabilize naturally^1^. The bioactivity of pectin polysaccharides includes numerous pharmacological uses such as antibacterial, anti-inflammatory, immunoregulatory, and antioxidant properties^2^. Chitosan is a biopolymer made from chitin, which is a linear cationic polysaccharide found in the shells of marine animals including prawns, lobster, and crabs^3^. Due to its antibacterial activity, biodegradability, biocompatibility, low toxicity, high water permeability, and responsiveness to chemical changes, it has gained extensive use in the chemical, biomedical, and food industries^4^. Numerous fungi, harmful bacteria, and microbes that cause spoilage are prevented from growing by chitosan^5^. Chitosan possesses significant antimicrobial and antioxidant properties with a high potential to develop biodegradable active packaging. Chitosan nanoparticles have attracted a lot of attention and are employed in an array of products and applications as well as for extensive antibacterial functions^6^.

Polyvinyl alcohol (PVA), is an affordable synthetic polymer with biodegradable properties, that has established frequent use in food packaging due to its favorable characteristics^7^. PVA possesses a wide range of adjustable physicochemical properties, including viscosity, film-forming capability, emulsifying and dispersing properties, tensile strength, and flexibility. It demonstrates thermostability, adhesive qualities, and capacity to withstand various solvents^8^. In recent years, significant focus has been directed toward PVA as a biocompatible, non-cytotoxic, processable, and degradable polymer within the fields of biomaterial and biomedical research^9,10^.

To create a Polyelectrolyte Complex (PEC), pectin has been utilized as a cross-linking agent for cationic polymers like chitosan^11^. Pectin has been shown to form a PEC with chitosan more efficiently than other polyanionic polymers. At pH levels between 3 and 6, chitosan cross-linked with pectin has been demonstrated to have increased hydrophilicity, biocompatibility, and mechanical strength, making it particularly effective for tissue engineering and drug administration^12^. At low pH levels (pH < 2), pectin and chitosan may also interact through hydrogen bonding. Numerous investigations on the antibacterial properties of pectin reveal a common propensity for the creation of nanocomposites and nano-emulsions. Both have observable inhibitory effects, mostly on Gram-positive Staphylococcus aureus and Gram-negative Escherichia coli^13^. The study of current composites should be conducted more thoroughly because of the potential for developing natural antimicrobials.

The PECs are formed as a result of electrostatic interaction between the amino group of chitosan (NH_3_^+^) and the carboxyl group of pectin (COO^−^). Pectin-chitosan PECs exhibit many desired properties, including increased solubility, stability, biodegradability, biocompatibility^14^, mechanical strength, in vivo targeting^15^, stimulated release, and poroviscoelasticity, which are needed in a variety of applications, including drug delivery, bone tissue engineering^16^, and soft tissue regeneration^17,18^. Due to its numerous uses in the fields of waste-water treatment, the food and pharmaceutical industries, medication delivery, and tissue engineering, PEC preparation is becoming more and more popular. PECs made from natural polymers have special qualities that cannot be seen in their polymer counterparts and are non-toxic and biocompatible^19^. Another advantage of PECs is their capability to be deposited onto any kind of substrate with complex geometric shapes like threaded dental implants^20^.

The long-term stability of implants has been extensively investigated in the literature. The stability of the implant is affected by the level of inflammation that affects the soft tissues and results in the loss of supporting bone, escalating progressive damage to the cortical bone, and peri-implantitis^21,22^. Titanium-based implants frequently develop an infection, which can be avoided by reducing the attachment of microorganisms to the surface of implanted devices^23^. The majority of peri-implant and periodontal infections are brought on by the streptococcus species, which frequently colonize implant surfaces^24,25^.

Despite the availability of numerous biomaterials, a perfect biomaterial with good surface properties and biocompatibility for clinical applications is hard to come by. Before deciding on using these new dental materials in clinical settings, the advantages and disadvantages of each should be evaluated in light of materials science principles and knowledge. PECs are the subject of considerable research, and as a result, new treatment approaches for dental applications have emerged. Therefore, the main objective of this study was to identify the ideal ratio of pectin-chitosan PCPC for use as a coating on titanium dental implants. In the present study, pectin and chitosan PEC solutions were prepared in different ratios of 1:1, 2:1, 1:2, 1:3, and 3:1 using the electrospraying technique, after adding polyvinyl alcohol (PVA). The physical and mechanical, and topographical features of the PCPCs layer using FESEM, AFM, wettability, cross-cut adhesion, and microhardness followed by the evaluation of the antibacterial characteristics were assessed.

## Material and methods

### Materials

Chitosan from shrimp shells (≥ 75% deacetylated, medium M_W_ 190–310 kDa) was purchased from (Sigma-Aldrich, USA). Pectin from the citrus peel (Galacturonic acid ≥ 74.0%, average M_W_ 485 kDa) dried basis was purchased from (Sigma-Aldrich, USA). PVA (98% hydrolyzed, average Mw 13–23 kDa) and acetic acid were purchased from Merck, Germany. All other experimental reagents used were analytical grade reagents; the experimental water was deionized water (DW). Commercially pure titanium discs (18 mm diameter, and 2 mm thickness) were prepared from titanium rods after cutting using a wire cut machine (DK 7735, China).

### Samples preparation

**Figure 1:**
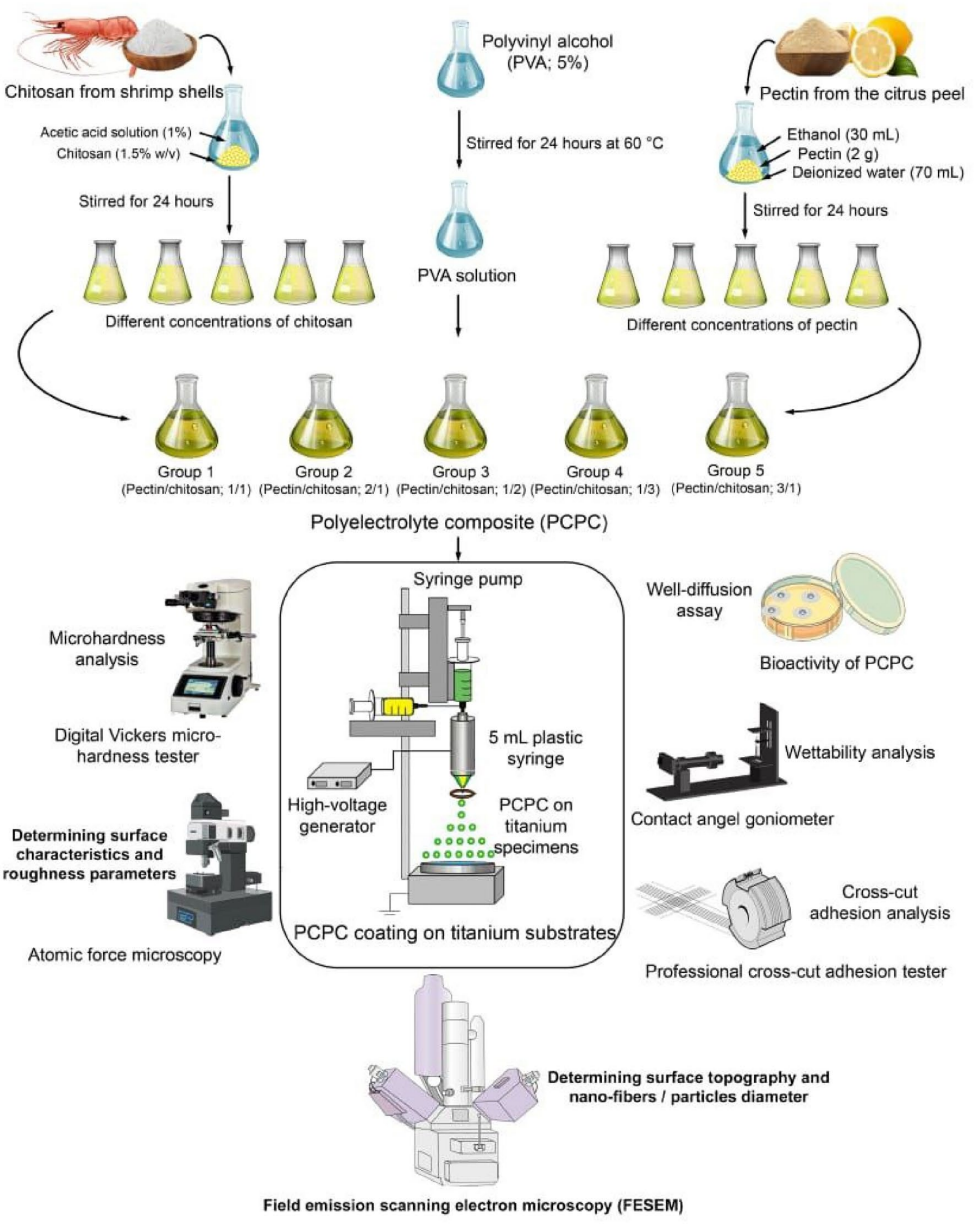
The experimental setup of the study.

As depicted in (Fig. 1), pectin (2.0 g) was mixed with ethanol (30 mL) and then with deionized water (70 mL), and after that stirred for 24 h to achieve complete dissolution. The chitosan sample (1.5% w/v) was dissolved in an acetic acid solution (1%) and stirred for 24 h to achieve complete dissolution. PVA solution (5%) was prepared in deionized water and stirred for 24 h at 60 °C to achieve complete dissolution. Around 30, 40, 20, 15, and 45 mL of pectin solutions were added to 30, 20, 40, 45, and 15 mL of chitosan solutions respectively with 30 mL of PVA added to the final solution (total volume 90 mL) and stirred at room temperature for 24 h. Thus, obtaining different pectin-chitosan ratios (1:1, 2:1, 1:2, 1:3, and 3:1). PVA was added only to improve the processability of the chitosan-based polymer with a fixed ratio in all five groups. Totally there were five groups for the pectin-chitosan polyelectrolyte composite (PCPC). (Table 1) illustrates the experimental design for preparing different groups with a combination of the mentioned three solutions. Following the characterization analyses, the two most appropriate ratios were selected in accordance with the current experimental study.

**Table 1:** Preparation and combination of three solutions.

Groups	Pectin (mL)	Chitosan (mL)	PVA (mL)	Ratio
Group 1	30	30	30	1:1
Group 2	40	20	30	2:1
Group 3	20	40	30	1:2
Group 4	15	45	30	1:3
Group 5	45	15	30	3:1

Commercially pure Titanium CpTi grade II (ASTM B348) was utilized as the substrate for coating. The titanium rod was cut into circular discs with dimensions of 18 mm in diameter and 2 mm in thickness using a wire cut machine (DK 7735, China). The specimen cutting was made according to the standard ASM volume(9)^26^. The disc samples were ground using SiC grinding paper grit 500#, then the samples were cleaned using ethanol 96% (Scharlau, Spain) using ultrasonic cleaner (KQ 3200, China) for 20 min twice. Then put aside to dry at ambient temperature.

### Electrospraying of pectin-chitosan polyelectrolyte composite coating

The electrospraying system consists of a high-voltage generator (Ya-Feng Technology Co., Taiwan) which was set at 20 kV. The prepared solutions were placed in a 5 mL plastic syringe fitted with a 25-gauge stainless steel needle. The syringe was filled with a polymer solution and placed on a syringe pump (KD Scientific, MA, USA). The distance between the collector and the needle was 10 cm and the flow rate of the solution was set at 1 mL/h. The experiments were carried out at room temperature in a transparent closed box with relative humidity ranging between 40 to 60%. The time of the procedure was fixed for an hour, after that, the produced PCPC on titanium specimens was left aside for 24 h in ambient conditions to evaporate excess acid and water prior to further analyses.

### Five-coated groups analysis using FESEM

The coated titanium samples were sputtered with gold before the morphologies of each specimen were examined by FESEM (Inspect TM F50, FEI USA). FESEM was used to diagnose the phases and nano-fibers/particles, diameters of nano-fibers/particles, and distribution of samples as well as to characterize the morphology of the prepared samples.

### Five-coated groups analysis using AFM

Atomic force microscopy (AFM) relies on the scanning technique and provides a high-resolution 3D image from the surface of the sample. It is used for determining surface morphology, roughness, topography, and particle size distribution. An atomic force microscope is able to detect both conductive and nonconductive surfaces on the atomic scale. A sharp tip at the end of the cantilever is in contact with the surface of the development and the sample is displaced with piezoelectric scanners. The force on the tip causes deflection to be measured with tunneling capacitive or optical detectors, and the standard pressure applied to the joint is zero (to prevent any surface deformation). The surface roughness and mean diameters of each specimen with a scan area of 10 µm × 10 µm were measured by AFM (NaioAFM 2022 model, Nanosurf AG, Switzerland). A sample was mounted on double-sided tape, the AFM probe used for measurements with tapping mode was a gold reflective coating on the tip side of the cantilever (Tap190GD-G), and the detector side was coated with 70 nm of gold. The force constant of the cantilever was in the range of 28–75 N/m, while the probe specification was a beam shape, length 225 µm, width 38 µm, and resonance frequency 190 kHz.

### Wettability analysis of the five-coated groups

The contact angle measuring device was performed with a goniometer (CAM 110, Creating Nano Technologies, Taiwan). It was utilized to measure the water contact angle of the coated discs with different coating ratios of PCPC and compared with the control uncoated disc in order to select the best two ratios based on the hydrophilicity. Before the wettability tests, the discs were immersed in acetone and then in isopropyl alcohol for five minutes each and then dried at room temperature. The specimens were handled with forceps and gloved hands to avoid the transfer of oil and contamination from the skin to the specimens. The water contact angle was measured by a special tool attached to a digital camera to offer a picture after applying a drop of DW on an intended surface with a small syringe. A 30-s interval was needed to capture an image after applying a drop of liquid on the intended surface, the procedure was implemented at an ambient temperature. Since the wetting properties are very important in implantable material and considered an indicator for future good osseointegration, so surface wettability test (water contact angle test) was used to measure the amount of the PCPC wettability for the five tested groups. The disc with low contact angle measurement (high wetting surface) was elected from the coated specimens.

### Cross-cut adhesion analysis of the five-coated groups

**Figure 2:**
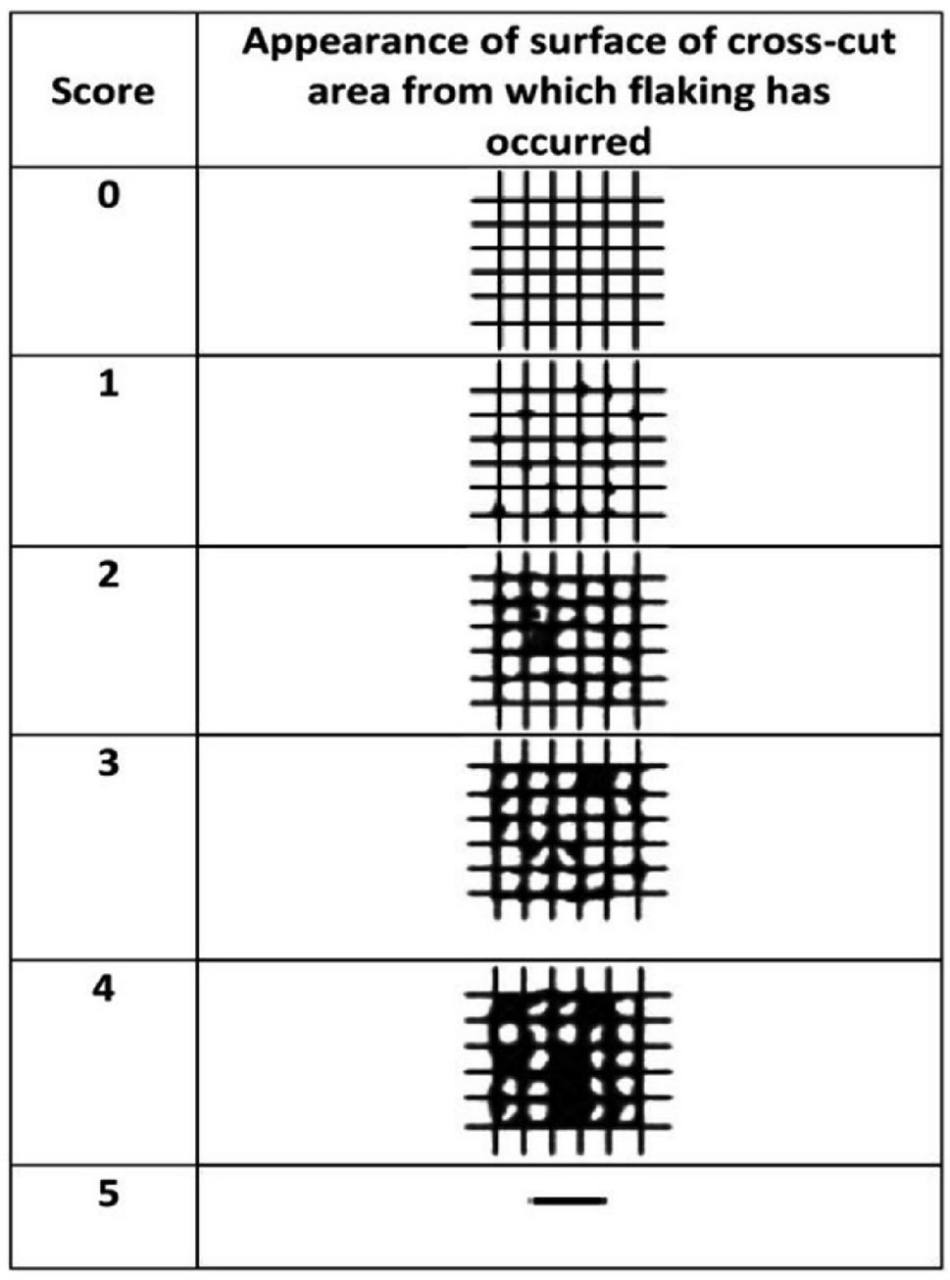
Classification of adhesion results following a 0–5 scale according to standards. Score 0: 0%, Score 1: 5%, Score 2: 5–15%, Score 3: 15–35%, Score 4: 35–65%, Score 5 > 65%.

The International Standard specifies a test method for assessing the resistance of coatings to separation from substrates when a right-angle lattice pattern is cut into the coating, penetrating through to the substrate. The property determined by this empirical test procedure depends, among other factors, on the adhesion of the coating to either the preceding coat or the substrate. This procedure is not to be regarded, however, as a means of measuring adhesion. The cross-cut test is not suitable for coatings of total thicknesses greater than 250 μm. This test was accomplished blindly by an operator for all the experimental groups with the device (professional cross-cut adhesion tester, ASTM D3359 ISO 2409:2013, Qualtech Industry Products Co., Ltd, China). The cutting tool with the blade normal (perpendicular) to the test panel surface was used. With uniform pressure on the cutting tool and using the appropriate spacing guide six cuts in the coating at a uniform cutting rate by moving the cutting tool in the direction approaching the operator was made. Each cut continued beyond the outermost of the cut perpendicular to it for approximately 1 mm to 2 mm. All cuts marked or scratched the substrate. The depth of indentation into the substrate was as low as possible. In (Fig. 2), a six-step classification is given for the illustration of adhesion outcomes determination after tape application. The first three steps are satisfactory for general purposes and are to be used when a pass/fail assessment is required. After that, the center of the tape was placed over the produced lattice in a direction parallel to one set of cuts, and the tape smoothed into place over the area of the lattice. To ensure good contact with the coating, the tape rubbed firmly with a fingertip or fingernail. Within 5 min after applying the tape, the tape was removed by grasping the free end and pulling it off steadily in 0.5 s to 1.0 s at an angle that is as close as possible to 60° and the outcome has been evaluated for each specimen.

### Microhardness analysis of the five-coated groups

Digital Vickers micro-hardness tester (Digital Microhardness Tester, HVS-1000/HVS-1000Z, Laryee Technology Co., Ltd, China) was used to record the microhardness of the uncoated and coated titanium discs of the five groups according to (ASTM E92-82, 1997), for 15 s 300 g load was applied to the surface of the discs by using Vickers indenter that joins optical microscopy. An average of 5 different readings was measured from each specimen in the five groups and compared with the uncoated control group.

### Antibacterial analysis of the five-coated groups

Cultures of total anaerobic bacteria were obtained by means of swab samples from 10 patients who were diagnosed with clinical implant failure and bone loss at the College of Dentistry, University of Baghdad, Iraq. The study was approved by the research ethics committee of the College of Dentistry, University of Baghdad, with registration number 790223. All methods were performed in accordance with the relevant guidelines and regulations. Informed consent was obtained from all subjects. The cultures were inoculated in an anaerobic chamber with 85% of N_2_, 10% H_2_, and 5% CO_2_ at 37 °C on blood agar plates for 48 h.

The antibacterial activity of the five experimental groups was tested. Bacteria were incubated in an anaerobic jar in Luria–Bertani broth media (Himedia, Mumbai, India) for 24 h. A colony-forming unit (CFU) of each bacterium was adjusted to 0.5 McFarland standards (approximately 1.5 × 10^8^ CFU/mL), and wells were made in Muller-Hinton agar media (Himedia, Mumbai, India). Cultures of bacteria with positive and negative controls were inoculated in the plates. Five groups of PCPC (1:1, 2:1, 1:2, 1:3, 3:1) were poured on marked wells and incubated anaerobically at 37 °C for 24 h. The zone of inhibition (clear zone of no growth of bacteria around each well) was measured in mm using a Vernier caliper. The minimum bacterial concentration (MBC) of the five experimental groups was determined by the broth microdilution method. 100 μ of 0.5 McFarland’s bacterial suspension denotes 10^8^ cells/mL was anaerobically inoculated into each Muller-Hinton agar plate. Then the five different ratios of PCPC were added and anaerobically incubated for 24 h at 37 °C.

### Statistical analysis

The statistical analysis was performed using Statistical Package for Social Science (SPSS version 27, Chicago in press, Illinois, USA). The experiments and measurements were repeated five times each, and the results were reported as means and standard deviations. For the bar charts graphical illustration, GraphPad Prism software (GraphPad Software, version 8.0.2, Inc., USA) was used.

## Results

### FESEM analysis

**Figure 3:**
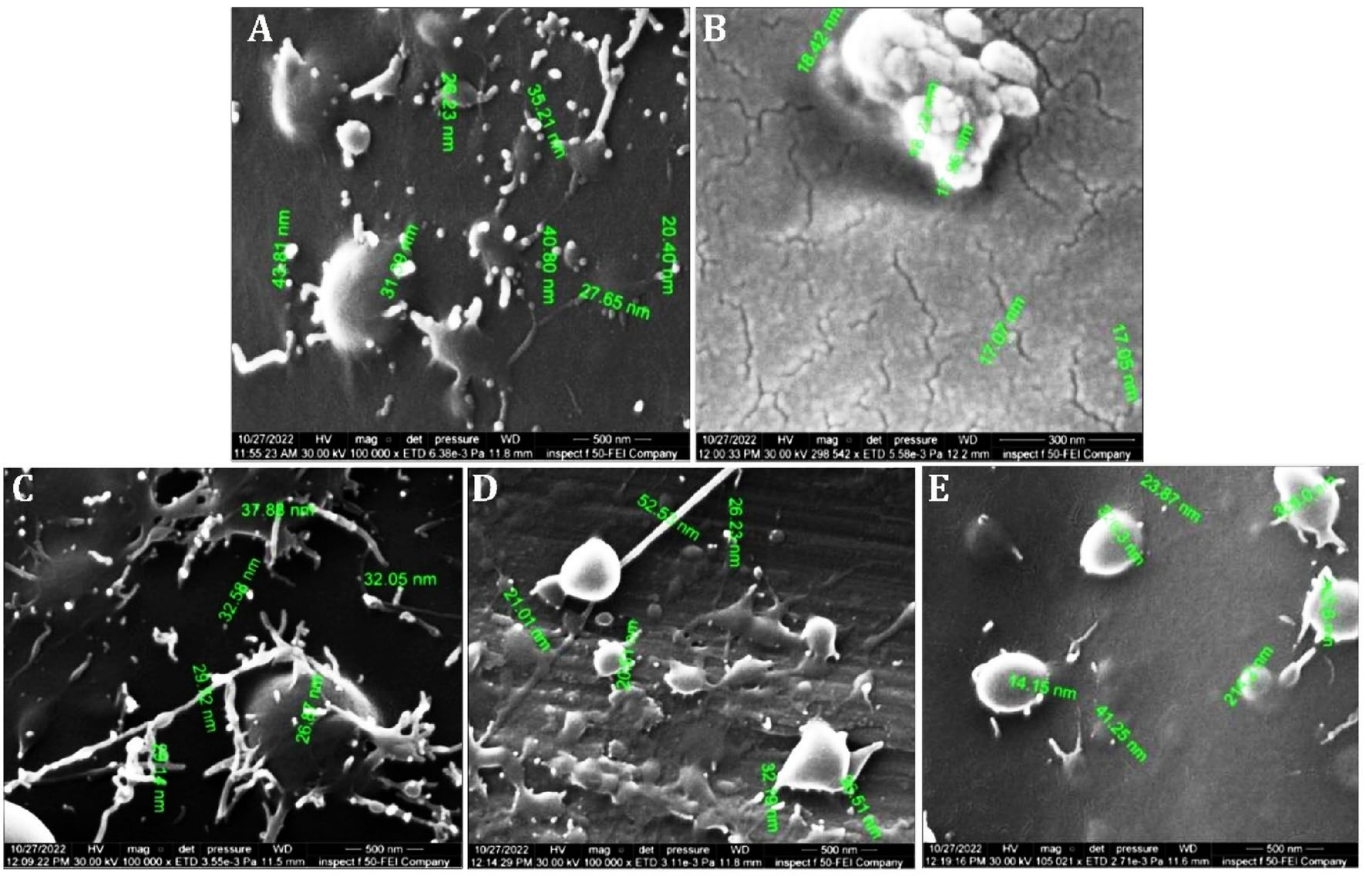
FESEM characterization of different CpTi substrates: (**A**) Coated substrate with group 1, (**B**) Coated substrate with group 2, (**C**) Coated substrate with group 3, (**D**) Coated substrate with group 4, (**E**) Coated substrate with group 5.

The morphological properties of the PCPC coating on titanium substrates were revealed by the FESEM as shown in (Fig. 3) for the five experimental groups. It was observed that the surface possesses irregular roughness and coverage. Groups 1, 3, and 4 demonstrated the formation of nanofibers and nanoparticles between 26 to 52 nm (Fig. 3A–C). Groups 2 and 5 had the least coating coverage and the largest particle size (Fig. 3B,E). Groups 3 and 4 demonstrated the combination of harmonized nano-spherical particles and nanofibers offering the best coating on the substrate surface (Fig. 3C,D).

### AFM

**Figure 4:**
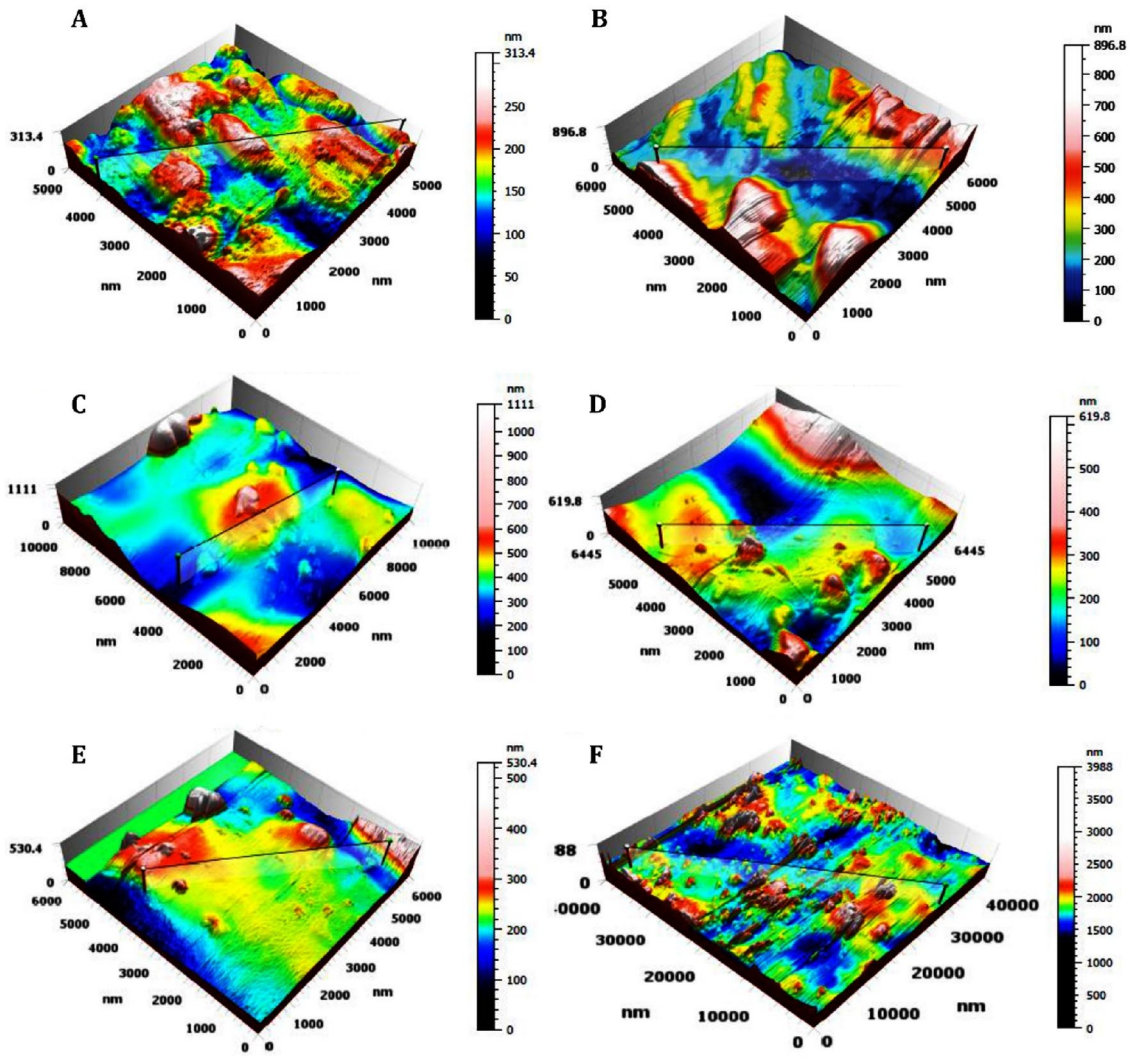
3D Illustration of AFM on different CpTi substrates: (**A**) Control, (**B**) Coated substrate with group 1, (**C**) Coated substrate with group 2, (**D**) Coated substrate with group 3, (**E**) Coated substrate with group 4, (**F**) Coated substrate with group 5.

The three-dimensional AFM images for the surface of control CpTi and the five experimental coated groups express the results obtained from the atomic force microscopy (Fig. 4). The color bar legends (right side of the image) beside the image are scales for *z*-values, which represent the height of the scanned samples. The morphologies were similar for the coated groups but markedly different from the CpTi control group. Figure 4A presents the images of the control sample with no coating. It is evident that the surface was relatively flat with low roughness parameters.

**Figure 5:**
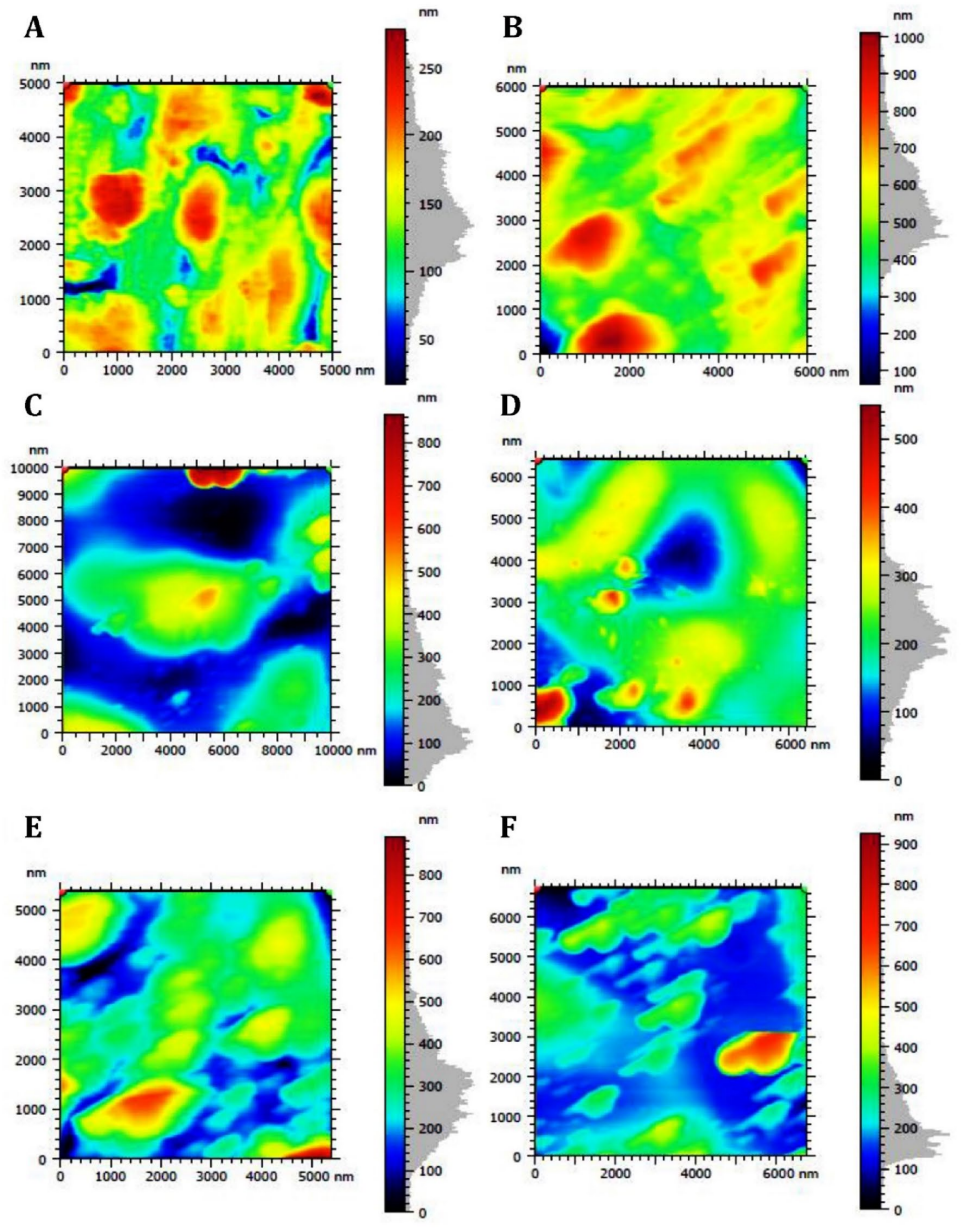
2D Illustration of AFM on different CpTi substrates: (**A**) Control, (**B**) Coated substrate with group 1, (**C**) Coated substrate with group 2, (**D**) Coated substrate with group 3, (**E**) Coated substrate with group 4, (**F**) Coated substrate with group 5.

In (Fig. 5), the roughness variations for the different ratios of coatings applied. The control group (Fig. 5A) had a few small beads with apparently more homogenous topography. Overall, the scans revealed more uneven surfaces containing different features for the coated substrates.

Table 2 presents quantitative results obtained from the AFM analysis. Groups 1 and 4 showed the highest roughness levels with varying coverage of the substrate surface. The highest coating coverage was achieved in Group 1. In contrast, Group 5 showed the largest diameter of deposited spherical particles.

**Table 2:** Roughness parameters for uncoated and coated CpTi substrates.

Roughness parameters								
Groups		Coverage %	Arithmetic mean height (Sa) nm	Max height (Sz) nm	Root-mean square height (Sq) nm	Mean diameter nm	Particles/mm^2^	Z-max mean nm
Control	NA	NA	10.02	132.5	13.34	101.0	1,480,000	661
Group 1	1:1	43.2	52.09	553.8	67.34	200.8	5,583,333	2066
Group 2	2:1	4.58	24.01	852.1	44.61	104.5	1,910,000	10,022
Group 3	1:2	20.7	23.33	372.4	35.11	90.9	8,786,284	2088
Group 4	1:3	34.7	55.43	681.1	73.35	80.5	16,333,333	915
Group 5	3:1	9.46	44.65	912.1	69.20	374.0	415,000	23,192

### Wettability analysis

**Figure 6:**
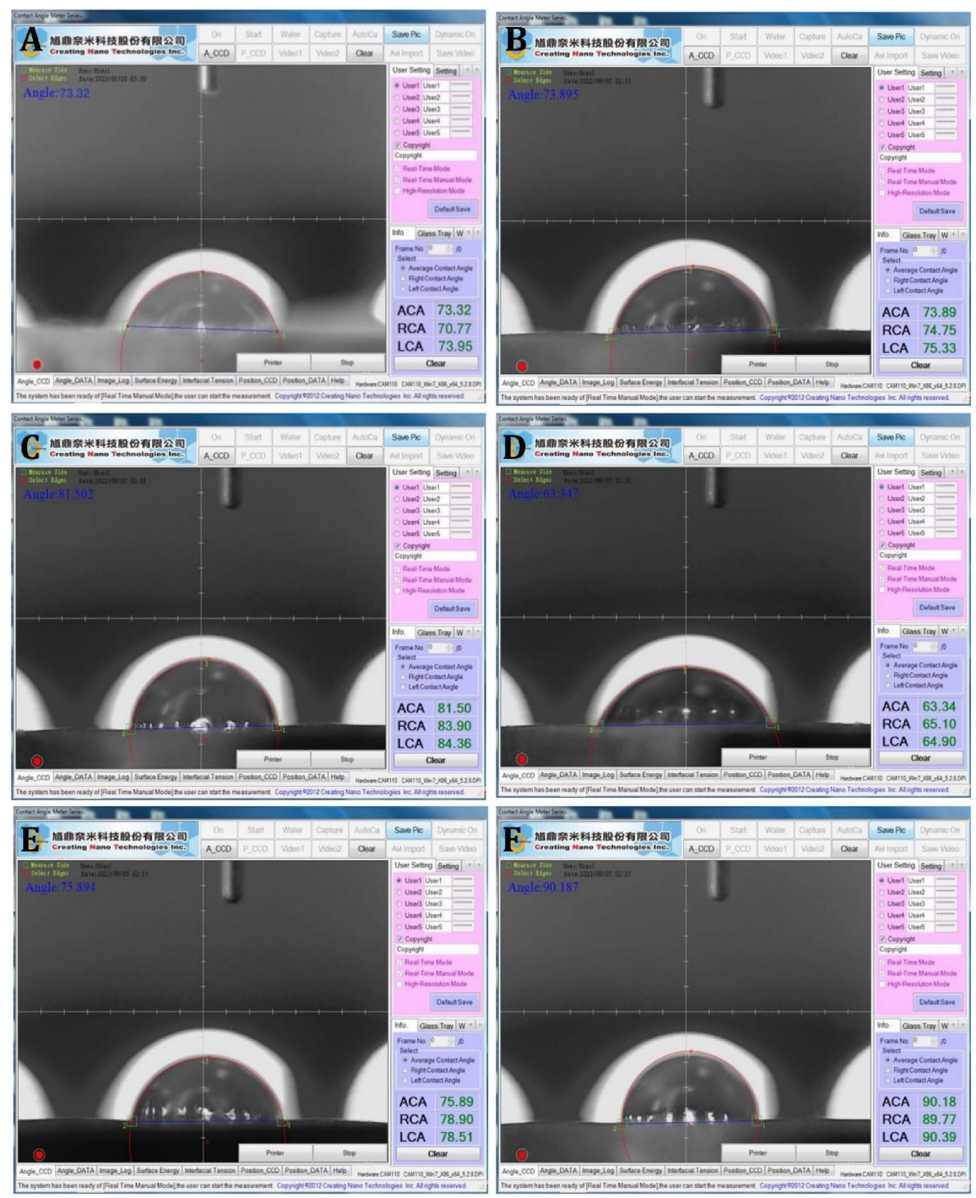
Illustration of contact angle test measurements on different CpTi substrates: (**A**) Control, (**B**) coated substrate with group 1, (**C**) coated substrate with group 2, (**D**) coated substrate with group 3, (**E**) coated substrate with group 4, (**F**) coated substrate with group 5.

**Figure 7:**
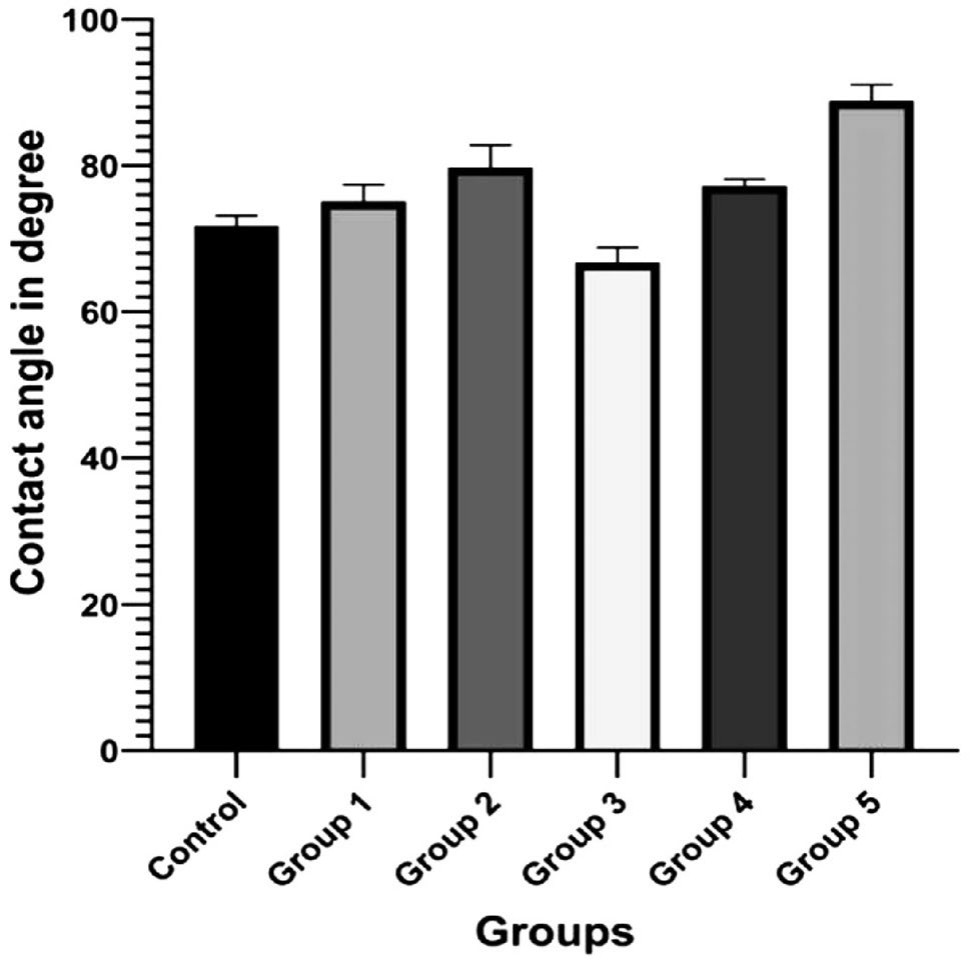
Illustration of contact angle test on different CpTi substrates.

According to the wettability results, the water contact angles of the five groups of PCPC and the control CpTi were between 66.73° and 88.86° with Group 3 displaying the lowest water contact angle. The results of the wettability test performed with the measurement of the contact angles are presented in (Fig. 6). Five separate readings were taken for each group and their means are reported in (Fig. 7). Decreasing the water contact angle leads to better wettability and increased protein adsorption on titanium surfaces^27^.

According to further statistical analysis considering the differences between the mean values of all groups (Table 3). Group 3 was the only group with a decreased contact angle than the control CpTi. The statistical analyses revealed a significant difference for Group 3 compared to all other tested groups.

**Table 3:** Statistical analysis between groups for wettability results.

Descriptive and statistical test of wettability among groups						
Groups	Mean	± SD	Minimum	Maximum	F	P value
Control	71.780	1.366	69.920	73.320	64.037	0.000 Sig.
G1	75.128	2.291	71.880	77.790		
G2	79.740	3.060	75.890	83.900		
G3	66.734	2.071	63.340	68.490		
G4	77.230	0.910	75.770	77.980		
G5	88.860	2.225	85.340	90.920		

Multiple comparisons of wettability among groups using Tukey HSD						
Groups		Mean difference	P value	Significance		
Control	G1	− 3.348	0.159	NS		
	G2	− 7.960	0.000	S		
	G3	5.046	0.010	S		
	G4	− 5.450	0.005	S		
	G5	− 17.080	0.000	S		
G1	G2	− 4.612	0.022	S		
	G3	8.394	0.000	S		
	G4	− 2.102	0.619	NS		
	G5	− 13.732	0.000	S		
G2	G3	13.006	0.000	S		
	G4	2.510	0.434	NS		
	G5	− 9.120	0.000	S		
G3	G4	− 10.496	0.000	S		
	G5	− 22.126	0.000	S		
G4	G5	− 11.630	0.000	S		

### Cross-cut adhesion

**Figure 8:**
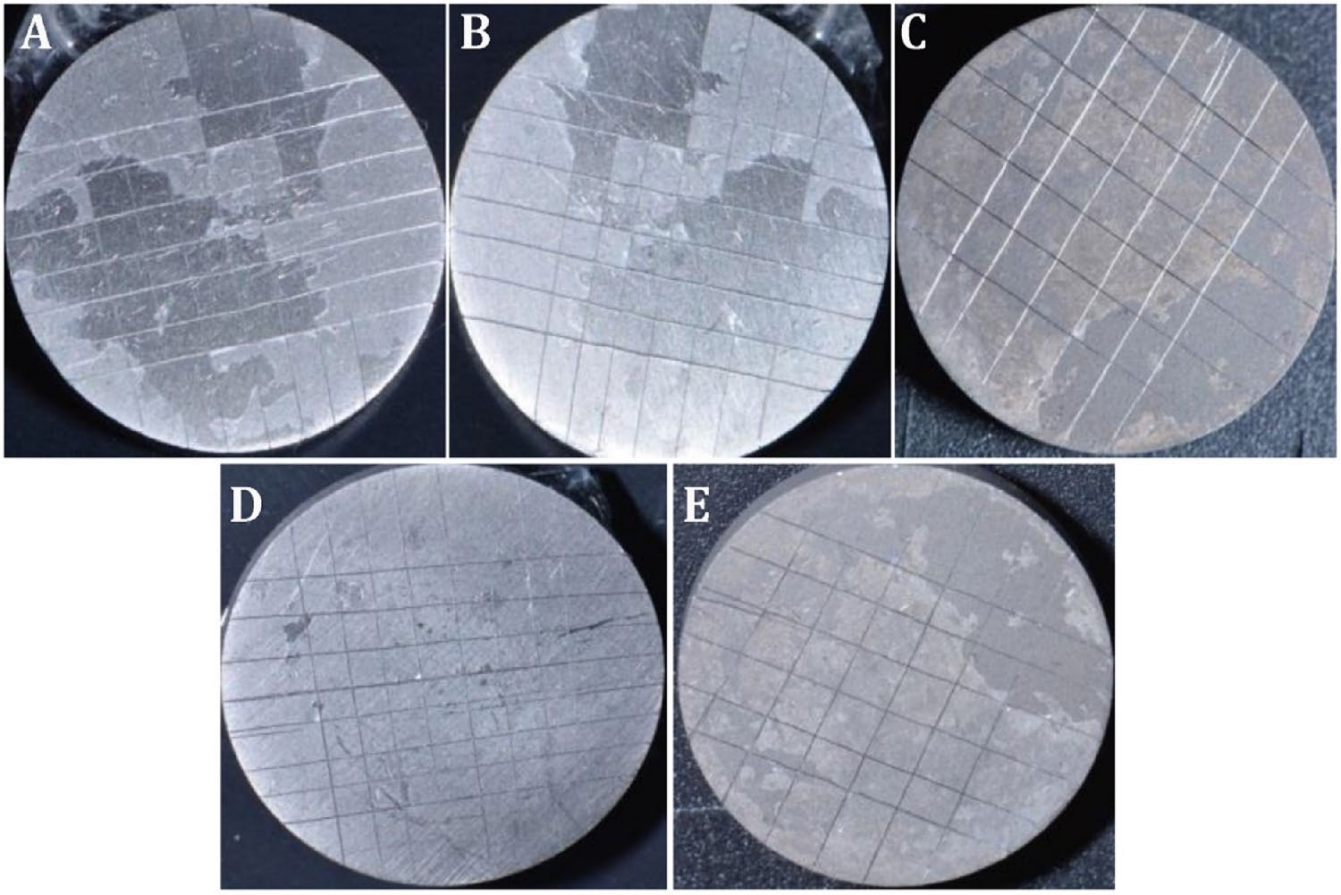
Illustration of cross-cut adhesion test on different CpTi coated substrates: (**A**) Coated substrate with group 1, (**B**) Coated substrate with group 2, (**C**) Coated substrate with group 3, (**D**) Coated substrate with group 4, (**E**) Coated substrate with group 5.

**Figure 9:**
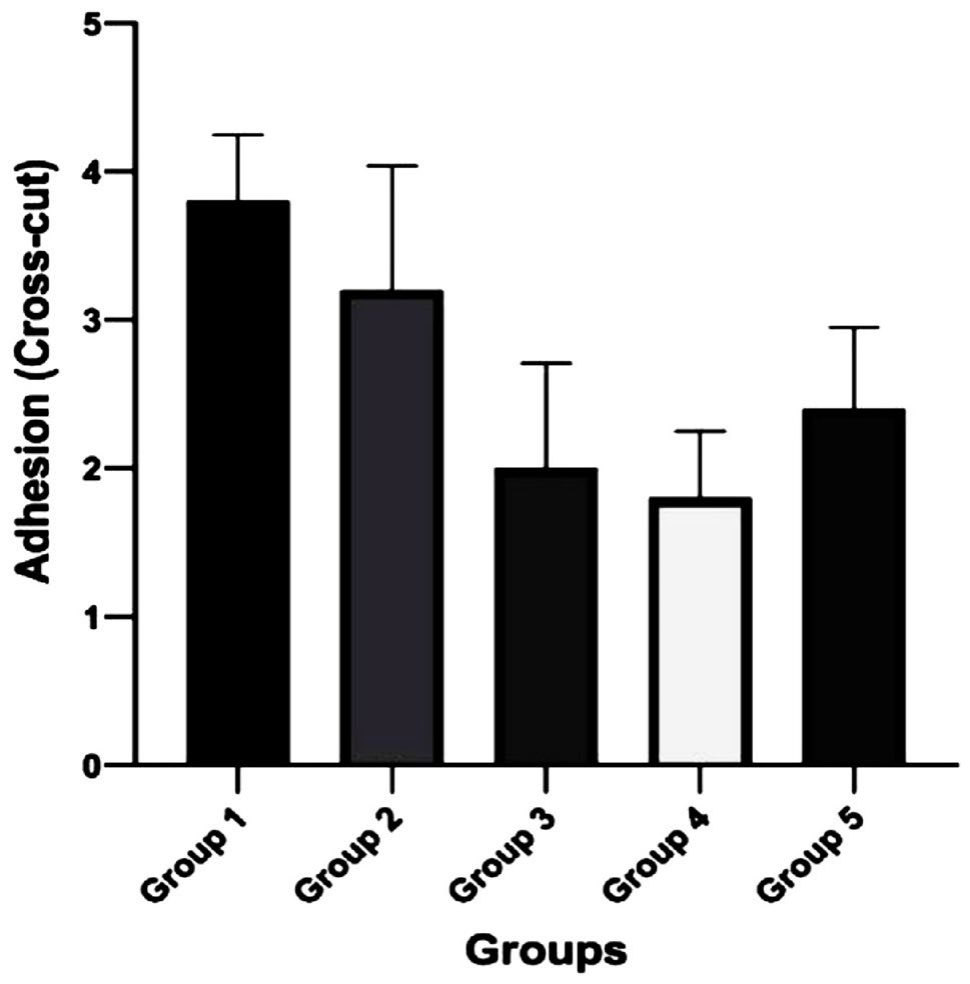
Cross-cut adhesion test results for different CpTi-coated substrates.

Adhesion is the resistance to separation of the coatings and substrates and is an important prerequisite for coating materials. Strong adhesion ensures better practicality and durability of coatings. Therefore, the evaluation of adhesion strength is crucial. According to the ASTM standard, the cross-cut test is a facile way to measure the level of adhesion. The performed cross-cut adhesion test is shown in (Fig. 8), while (Fig. 9) shows that the adhesion of coatings was greatly influenced by the content of the pectin in the PCPC coating, with lower levels of pectin presenting better adhesion. The highest level of adhesion value was obtained in group 4 and similar adhesion levels in group 3 (Fig. 8).

According to further statistical analysis considering the differences between the groups (Table 4). There was only a relevant significant difference between (group 1 and group 3), and (group 1 and group 4) in adhesion quality.

**Table 4:** Statistical analysis between groups for adhesion results.

Descriptive and statistical test of adhesion scores among groups						
Groups	Minimum	Maximum	Median	Mean rank	Kruskal–Wallis	P value
G1	3	4	4	21.30	15.585	0.000 Sig.
G2	2	4	3	17.20		
G3	1	3	2	8.40		
G4	1	2	2	6.70		
G5	2	3	2	11.40		

Multiple comparisons of adhesion among groups using multiple Wilcoxon sum rank test adjusted by Dunn–Bonferroni						
Groups		Mean rank difference	P value	Significance		
G1	G2	4.100	0.350	NS		
	G3	12.900	0.003	S		
	G4	14.600	0.001	S		
	G5	9.900	0.360	NS		
G2	G3	8.800	0.673	NS		
	G4	10.500	0.250	NS		
	G5	5.800	0.186	NS		
G3	G4	1.700	0.698	NS		
	G5	− 3.000	0.494	NS		
G4	G5	− 4.700	0.284	NS		

### Vickers hardness of the coating

**Figure 10:**
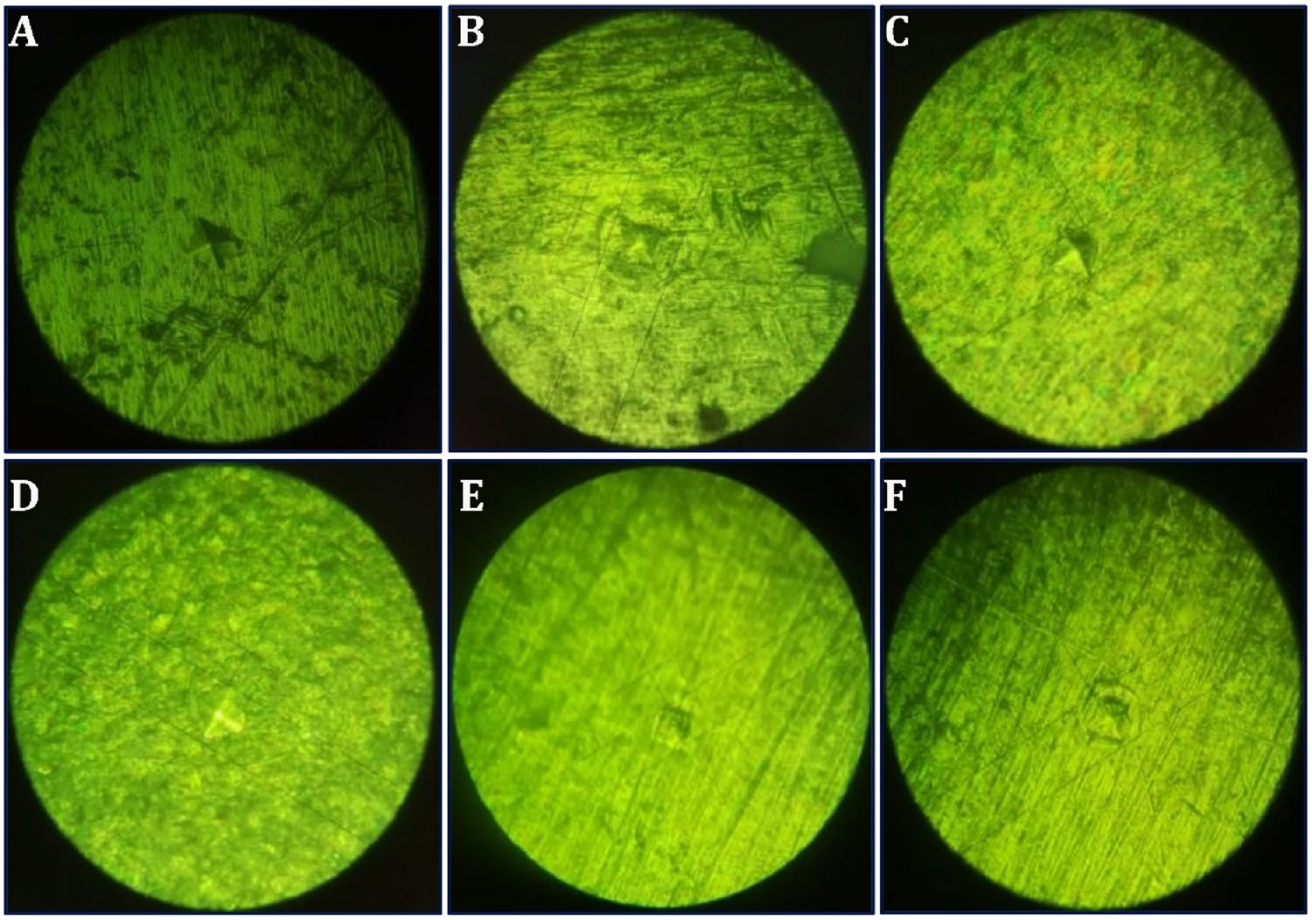
Illustration of VMH test on different CpTi substrates: (**A**) Control, (**B**) Coated substrate with group 1, (**C**) Coated substrate with group 2, (**D**) Coated substrate with group 3, (**E**) Coated substrate with group 4, (**F**) Coated substrate with group.

**Figure 11:**
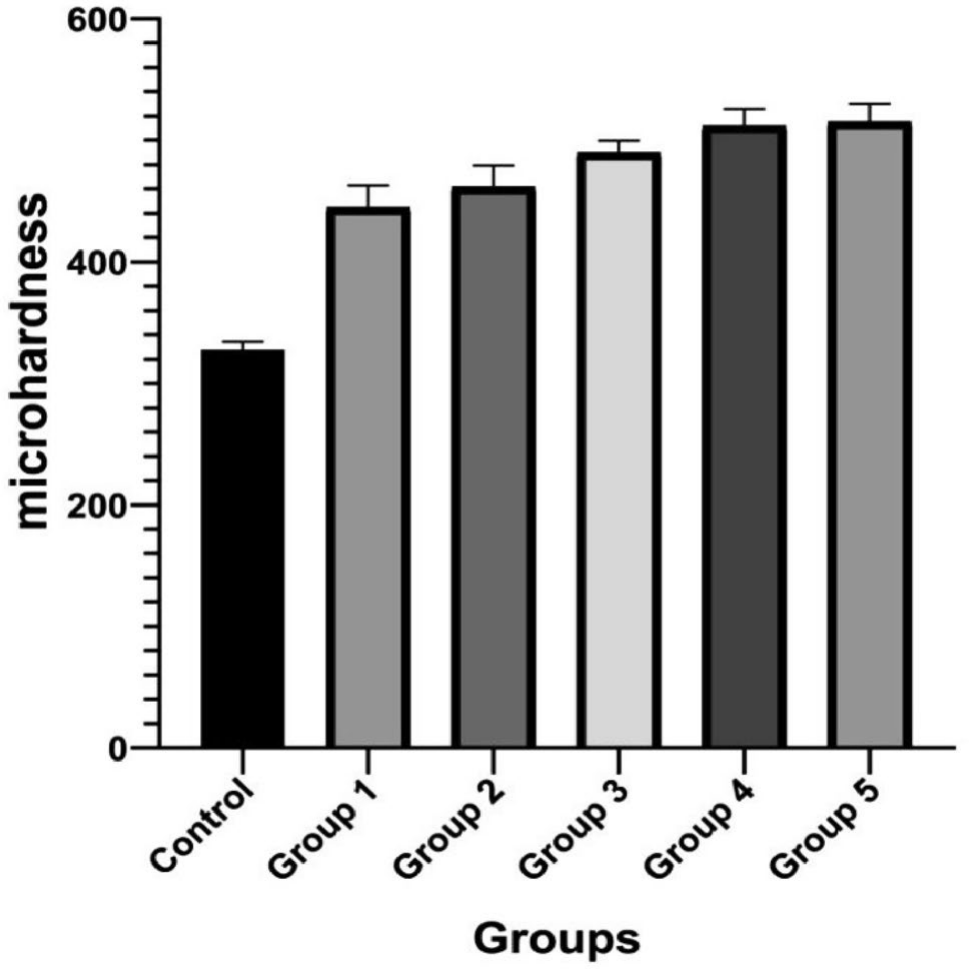
Vickers Microhardness test results for different CpTi substrates.

The mean values of the hardness of the different groups of the coating were higher than the control titanium discs. Figure 10 demonstrates the indentation created by the Vickers diamond indenter on each tested disc. According to (Fig. 11), the mean hardness value was the lowest for the control and the highest for Group 4 (1:3) and Group 5 (3:1). The strong link between the pectin and chitosan molecules and the flexible nature of the polyelectrolyte complex increases the HV values for the coated titanium discs.

According to further statistical analysis observing the differences between the experimental groups. All groups had highly relevant significant mean differences compared to the control CpTi group. Meanwhile, there was irrelevant significant difference between groups 3, 4, and 5 regarding the hardness values (Table 5).

**Table 5:** Statistical analysis between groups for microhardness results.

Descriptive and statistical test of surface microhardness among groups						
Groups	Mean	± SD	Minimum	Maximum	F	P value
Control	327.560	6.611	320.200	337.200	124.291	0.000 Sig.
G1	445.080	17.867	417.600	461.300		
G2	461.740	17.699	445.200	488.400		
G3	490.160	9.392	476.900	499.500		
G4	512.140	13.850	494.800	531.500		
G5	515.400	14.974	490.700	529.700		

Multiple comparisons of surface microhardness among groups using Tukey HSD						
Groups		Mean difference	p value	Significance		
Control	G1	− 117.520	0.000	S		
	G2	− 134.180	0.000	S		
	G3	− 162.600	0.000	S		
	G4	− 184.580	0.000	S		
	G5	− 187.840	0.000	S		
G1	G2	− 16.660	0.439	NS		
	G3	− 45.080	0.000	S		
	G4	− 67.060	0.000	S		
	G5	− 70.320	0.000	S		
G2	G3	− 28.420	0.039	S		
	G4	− 50.400	0.000	S		
	G5	− 53.660	0.000	S		
G3	G4	− 21.980	0.170	NS		
	G5	− 25.240	0.084	NS		
G4	G5	− 3.260	0.999	NS		

### Antibacterial activity

**Figure 12:**
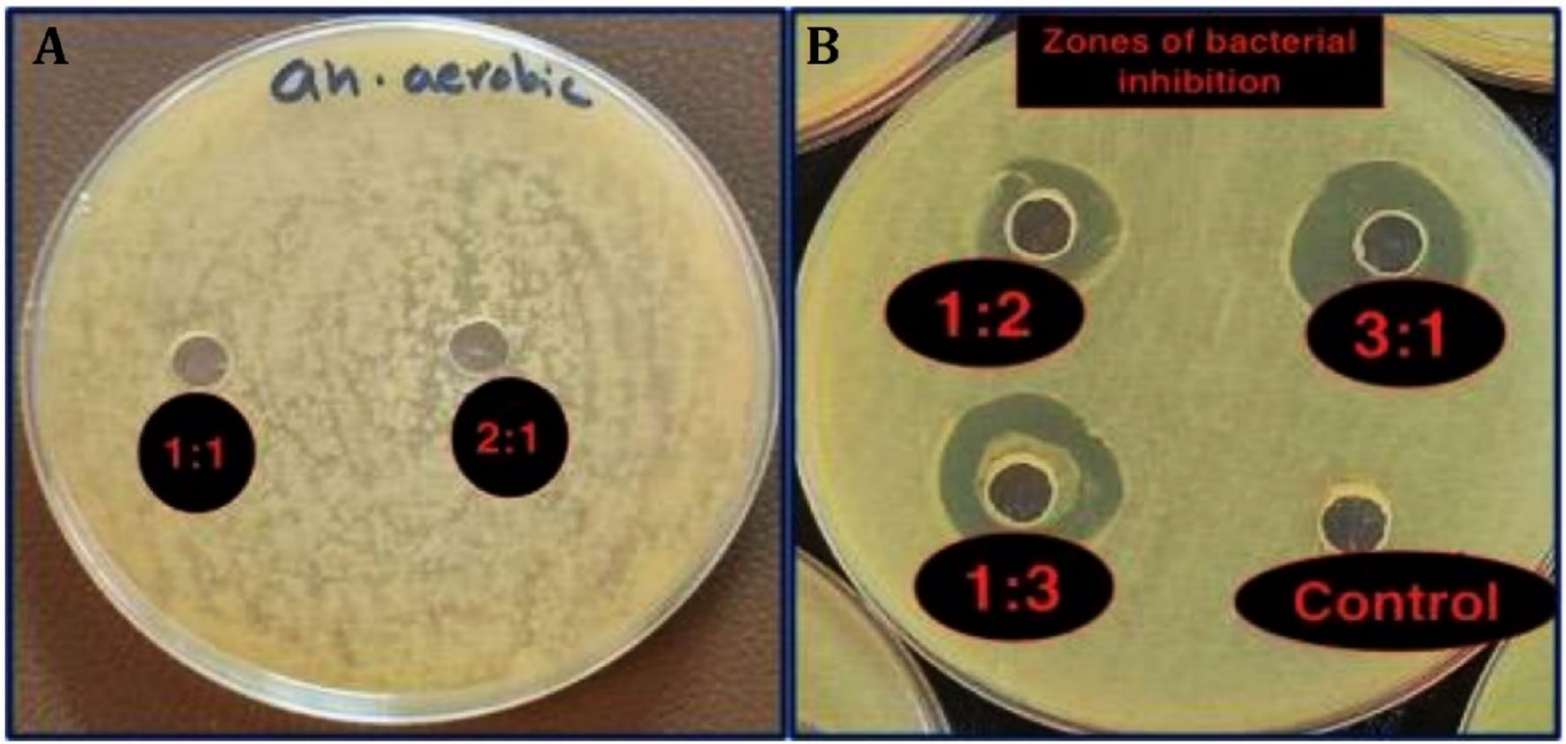
Antibacterial activity of the five groups against oral microorganisms, (**A**) Group 1, and group 2, (**B**) Control (bottom right), group 5 (bottom left), group 4 (top right), and group 3 (top left).

**Figure 13:**
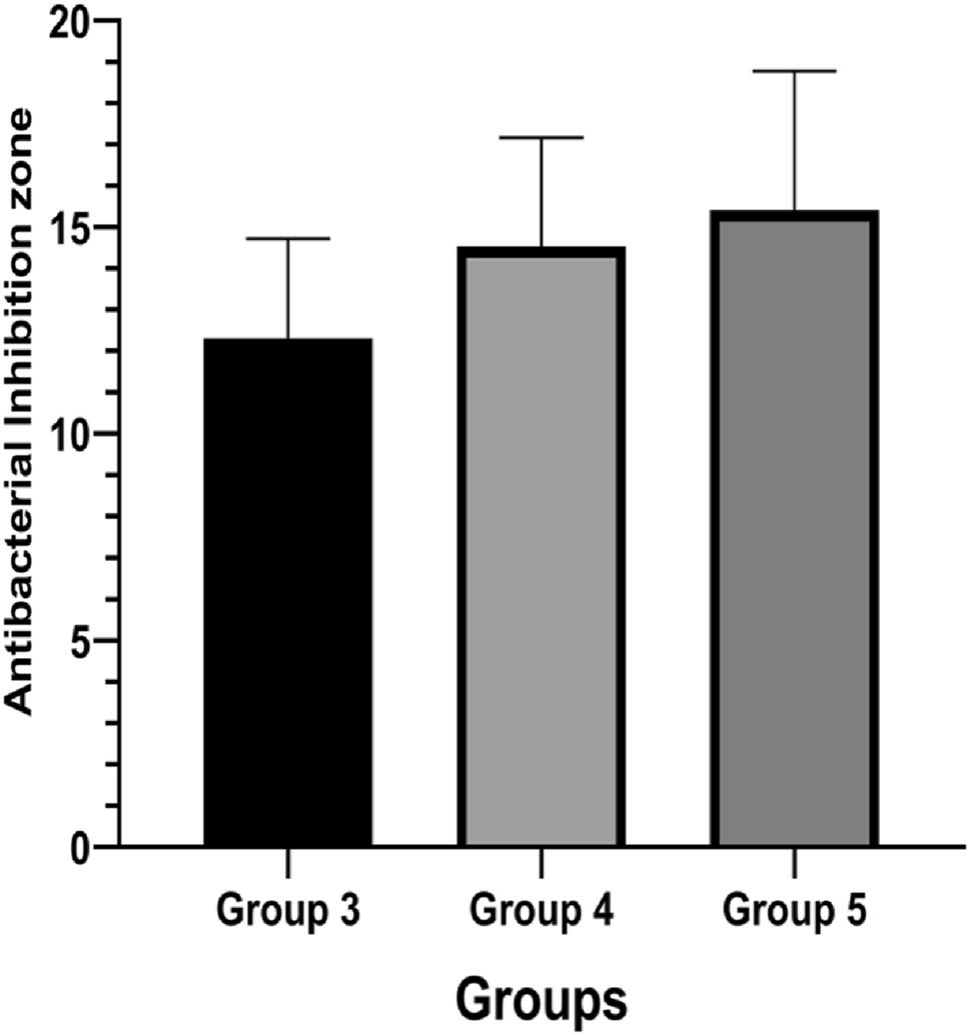
Antibacterial inhibition zone diameter (mm) for groups 3, 4, and 5.

The zone of inhibition test (also referred to as agar-well diffusion assay) is used to determine the susceptibility or resistance of pathogenic bacteria to antibacterial agents. A zone of inhibition (ZOI) is a clear circular around antimicrobial discs in which bacteria are unable to grow. Both ZOI and MBC investigations confirmed that PCPC can prevent anaerobic bacterial growth. The pectin-chitosan composite 3:1 ratio showed the largest inhibition zone, with no response to the ten isolates in Groups 1 (1:1) and 2 (2:1), which then have been excluded from the multiple comparisons as seen in (Fig. 12). Illustration of the mean values for the inhibition zone diameter for Groups 3, 4, and 5 are presented in (Fig. 13). Although, the analysis of variance revealed a borderline significant P value (0.057) among the three groups. The obtained results could indicate a lack of statistically relevant significant differences among the three experimental groups, as determined by the analysis of the zone of inhibition against oral microorganisms (Table 6).

**Table 6:** Statistical analysis between groups for zones of inhibition diameter.

Descriptive and statistical test of inhibition zone diameter among groups							
Groups	N	Mean	± SD	Minimum	Maximum	F	P value
G3	10	12.312	2.402	8.590	15.890	3.194	0.057
G4	10	14.538	2.626	10.570	18.330		
G5	10	15.413	3.368	9.200	20.230		

**Figure 14:**
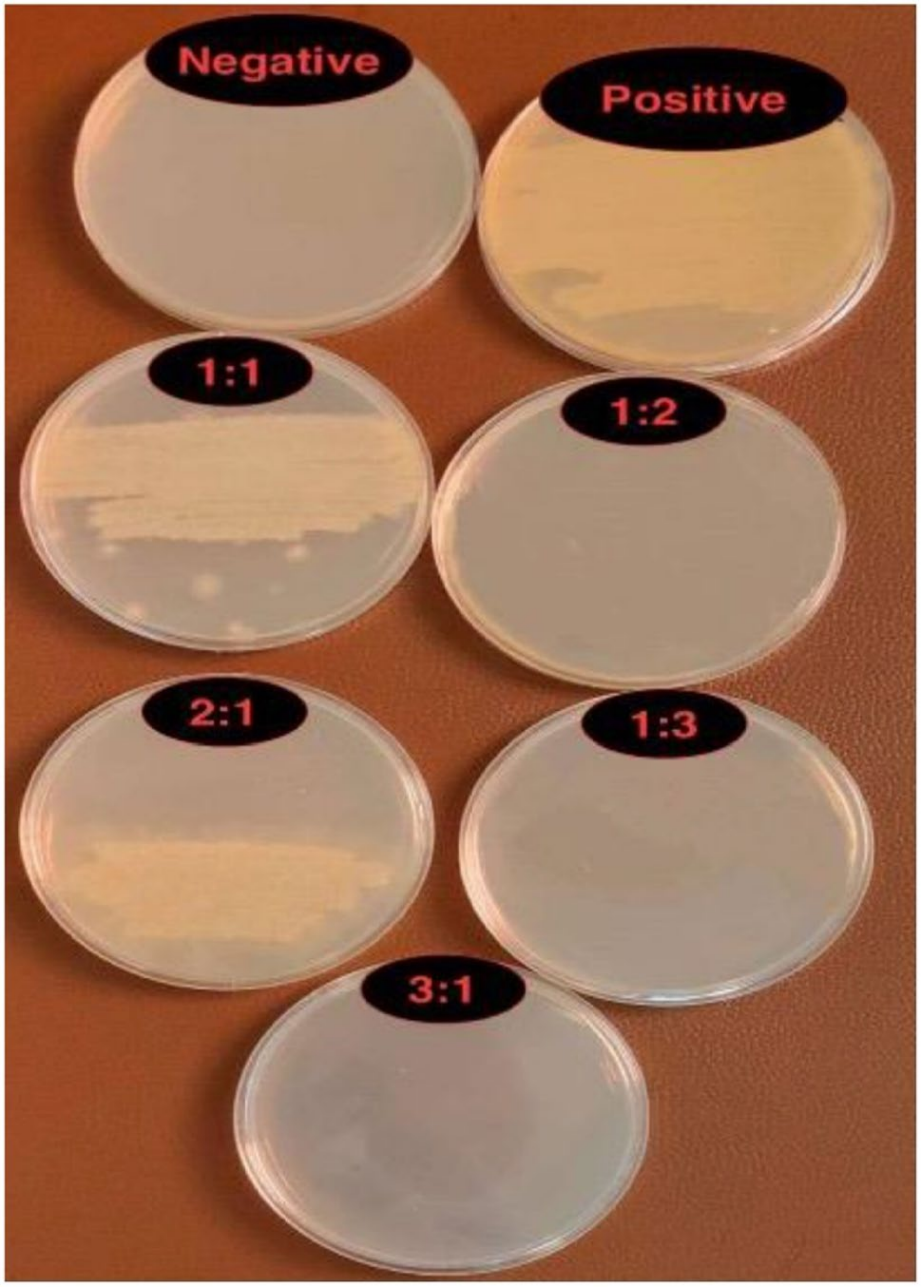
Minimum bactericidal concentration (MBC) for the five experimental groups.

The results of the minimum bactericidal concentration (MBC) assay revealed that the PCPC group 3 (1:2) exhibited no growth after re-culturing on MHA media. This finding indicates that group 3 possessed a bactericidal effect that effectively eliminated the microorganism (Fig. 14). These outcomes confirmed the results of the agar-well diffusion assay for antibacterial activity of the tested PCPC groups.

## Discussion

The present study aimed at developing a chitosan-pectin polyelectrolyte composite to be electrosprayed on a commercially pure titanium surface that could be used as a dental implant. Several ratios of chitosan and pectin have been proposed in the literature; however, they are usually in the gel state or in the sol state. Our study used the electrospraying method to create PCPC coatings in ratios of 1:1, 2:1, 1:2, 1:3, and 3:1 using PVA solution as a base. The physical and mechanical properties of the different ratios were evaluated. FESEM analysis revealed a combination of spherical particles and nanofibers. AFM analysis revealed higher surface roughness for Group 5 and homogenous coating for Group 1. Group 3 showed the lowest water contact angle of 66.7° and all PCPC coatings had higher Vickers hardness values compared to the control sample. According to the 0–5 scale of the cross-cut adhesion test, Groups 4 and 3 showed the best adhesion of the PCPC to the titanium substrates. Groups 3, 4, and 5 showed antibacterial properties with a significant zone of inhibitions compared to the control. Overall, Groups 3 and 4 showed the ideal morphological and mechanical properties with higher surface roughness, higher surface strength, increased hydrophilicity, and increased adhesion to the substrate surface.

Sousa et al. compared 1:1 and 2:1 pectin-chitosan PEC and found irregular, highly porous structural form in the SEM analysis. They described the coating to be from sheet-like to fibrous-like structure with the pores being interconnected^28^. Another author used scanning electron microscopy and observed the pectin-chitosan PEC complex in the ratio of 4:1 to have a flat and smooth PEC film surface indicating the mixture of pectin-chitosan to be a homogenous^29^. Martin et al. used multilayers of PEC made from pectin and chitosan. They applied 5 layers and 15 layers respectively on a glass surface to evaluate their mechanical and physical properties^30^. They found the root mean square roughness values for pectin-chitosan layers to be 1.1 and 3.5 respectively for the 5 layer and 15 layers. In our study, FESEM and AFM analyses showed similar results of irregular porous coating containing both nano-spherical particles and fibers. Groups 1, 3, and 4 were more sheet-like and homogenous with nano-particles incorporated. Simultaneously, it was observed that Groups 1 and 4 coatings exhibited higher levels of surface roughness. In contrast, groups 2 and 5 demonstrated the lowest coating coverage.

The contact angle of a material with water can exhibit hydrophilicity characteristics. The smaller the contact angle, the more hydrophilic the material would be. In previous research, pectin was usually deemed as a hydrophilic polysaccharide^31^. In contrast, pure chitosan produced a water contact angle of 86°. Looking at the combination of pectin-chitosan PEC, another study found that when pectin was added to chitosan, the contact angle decreased to about 45° for equal parts of pectin and chitosan coating, proposing increased hydrophilicity in the presence of pectin^32^. Ruei et al. added pectin and gum arabica to chitosan in varying ratios and found a similar contact angle decreased from 92.2° to 84.3° indicating that the addition of pectin and gum arabica improved the hydrophilicity of the PEC coatings ^33^. Alessandro et al. compared wettability between calcium-chitosan PEC and pectin-chitosan PEC, and found that the pectin-chitosan PEC was less hydrophilic with a 51° water contact angle. It was still more hydrophilic than the control surface which had a 62° contact angle^30^. The surface characteristics play an essential role in the establishment of interactions between surfaces and cells. More hydrophilic surfaces can provide microenvironments to promote cell adhesion, thereby promoting cell spreading, proliferation, and migration. Viktoryia also found similar results for PEC layers made from pectin and chitosan molecules. Their experiment found water contact angles less than 90°. They also found that the lowest contact angles were observed along with a high surface roughness^34^. Our study found a decreased water contact angle in Group 3 compared to the control CpTi with a 7% reduction. However, PVA is inherently hydrophilic, it was consistently incorporated at a fixed ratio with the main components, pectin, and chitosan, in all experimental groups in our study. This might elucidate why the contact angle did not significantly reduce when compared to the control CpTi. Gurzawska et al. in a systemic review, discovered an increased osteoblast attachment to pectin-coated surfaces with higher osteoblast differentiation and mineralization. This finding supports the idea that the polysaccharide coatings exhibit hydrophilic properties^35^.

The cross-cut test is a fast method to measure the adhesion of the coating on the CpTi substrate. However, when adhesion is too high, it is difficult to further evaluate the level of adhesion with greater precision. Additionally, during the cross-cut testing, ensuring complete consistency in the force applied to the tape and the speed at which it is pulled is difficult, which can result in deviations in the results. Rashidova et al., studied the PEC formed between chitosan and pectin in different ratios. They obtained pectin-chitosan interaction in a 2.0% acetic acid solution and observed that structural toughness depended on mixture composition^36^. Bigucci et al. found the best ionic interaction between pectin and chitosan for ratios of 1:1 and 3:7 at pH 5 of the solution^37^. Groups 3 and 4 showed the best adhesion results when compared with other tested groups. Since PVA was consistently included in all groups at the same ratio, it was not feasible to assess the specific impact of PVA on adhesive characteristics.

Mechanical integrity is a crucial quality for biomaterials used in load-bearing applications. A biomaterial having mechanical qualities that are either weak or powerful is not ideal since it could hinder tissue regeneration or harm nearby tissues. As a result, adequate mechanical strength is required to enable cellular processes such as cell adhesion, migration, and proliferation. Rajkumar et al. found that pure chitosan coatings on dental implants reduced the Vickers hardness of the implant surface^38^. Whereas another research found that pure pectin coating on implants increased the hardness of the implant surface^39^. Our study displayed an increase in Vickers hardness of the PCPC coatings applied to the titanium surface. The addition of both pectin and chitosan along with the synergistic addition of PVA to the coating, increased the fibers' size, resulting in a stronger and more mechanically robust coated layer due to the cross-linking effect of various combined molecules. Incorporating PVA into natural biopolymers aids as a plasticizing agent, reducing the brittleness while improving the coating toughness^40^.

In implant materials, preventing the initial attachment of bacteria along with promoting the growth of healthy tissue, are essential for the long-term success of dental implants. Several studies have shown that pectin and chitosan are not cytotoxic to human cells^28^. Martins looked at the in-vivo attachment of bacteria on pectin-chitosan PECs. They found significantly decreased attachment of *P. aeruginosa* and *S. aureus* bacteria on the PEC-coated surfaces compared to control samples^30^. Our study also showed a good zone of inhibition around the coated substrates with group 3 having a significant p-value when compared to other groups. The MBC test further verified that Groups 3, 4, and 5 had excellent results and prevented the growth of anaerobic microorganisms. Comparing the different groups in our studies, the ideal properties were for Groups 3 and 4. The ratios of 1:2 and 1:3, where chitosan was present in more quantities than pectin, and consistent PVA ratio were ideal for coating titanium implants. The two groups had higher surface roughness with homogenous nano-particles/fibers along with high surface coverage. Both groups had significantly higher surface strength, increased hydrophilicity, and increased adhesion to the titanium substrates. The antibacterial tests demonstrated sufficient ZOI around both groups of PCPC.

## Conclusions

The study highlights the benefits of PCPC's ability to form physically and structurally stable coatings on commercially pure titanium substrates. The PCPC coating's characteristics can be significantly impacted by the use of certain pectin-to-chitosan ratios. Groups 3 (1:2) and 4 (1:3) showed remarkable morphological and mechanical properties with better surface roughness, greater surface strength, improved hydrophilicity, improved adhesion to the substrate surface, and additionally demonstrated significant antibacterial properties. According to this accomplished in vitro study outcome, these particular PCPC ratios can be considered as an efficient coating of titanium dental implants after further animal studies assessment which will be investigated later.

### Limitations and future perspectives

The current study's limitations pertain to the in vitro analysis of the pectin-chitosan polyelectrolyte composite (PCPC) coating on commercial pure titanium substrates, focusing on a range of physical, mechanical, topographical, and antibacterial properties. A further limitation arises from the exclusive use of a metallic substrate; evaluating the coating on ceramic and polymer substrates to explore diverse characteristics would be beneficial. Additionally, although antibacterial assessments have been carried out, they are still preliminary. A comprehensive evaluation of the physical, chemical, and mechanical properties of the PCPC coating on implant screws is necessary to facilitate future animal studies, which are crucial for anticipating cellular responses and estimating the therapeutic efficacy of these coatings. Implementing the use of PCPC-coated implants in animal models that simulate physiological conditions including both healthy and compromised situations such as infections or poor bone quality over prolonged periods is vital for a thorough appraisal of implant coatings. To confirm the biocompatibility of these materials, cytotoxicity and sensitivity assays should be incorporated into both experimental and animal study designs. To establish clinical validation for the acceleration of bone osteointegration and the reduction of bacterial adhesion, further investigation is needed. Looking ahead, the application of PCPC coatings may be advanced through additive manufacturing or 3D printing technologies, enabling the production of customized implants tailored to patient-specific requirements after meticulous experimental and clinical evaluation.

## Data Availability

All data generated or analyzed during this study are included in this published article.
